# Diseases of the Eye

**Published:** 1876-10

**Authors:** 


DISEASES OF THE EYE. By Robert Brudenell Carter, F.R.C.S., Ophthalmic Surgeon to St. George's Hospital, Surgeon to the Royal South London Ophthalmic Hospital, Consulting Surgeon to the Gloucestershire Eye Institution, Hunterian Professor of Surgery and Pathology to the Royal College of Surgeons of England. With one hundred and twenty-four illustrations; edited with additions, and test types by John Green, M.D. Henry C. Lea, publisher. Philadelphia: 1876.

This is a handsome volume of five hundred pages, and presents, in a precise and practical manner, the principles of ophthalmology. The compendious arrangement of its subjects, and the explicitness of its elementary details, render it a superior text-book for students; while, in addition, the practical ideas and scientific illustrations adduced by the experienced author entitle it to a high grade as a book of reference. Commencing with a detailed anatomical and physiological description of the organ of sight, the means of examining the eyes, and the pathological appearances discovered therefrom, is followed by a comprehensive treatise on ophthalmic therapeutics and surgery. A systematized pathology of the more important diseases is next presented, with the appropriate and most approved treatment of each. There is no book in our library that we can more highly appreciate than this practical treatise on diseases of the eye.

